# Maintained anxiolytic effects of cannabidiol after treatment discontinuation in healthcare workers during the COVID-19 pandemic

**DOI:** 10.3389/fphar.2022.856846

**Published:** 2022-10-03

**Authors:** José Diogo S. Souza, Antonio W. Zuardi, Francisco S. Guimarães, Flávia de Lima Osório, Sonia Regina Loureiro, Alline Cristina Campos, Jaime E. C. Hallak, Rafael G. Dos Santos, Isabella Lara Machado Silveira, Karina Pereira-Lima, Julia Cozar Pacheco, Juliana Mayumi Ushirohira, Rafael Rinaldi Ferreira, Karla Cristinne Mancini Costa, Davi Silveira Scomparin, Franciele Franco Scarante, Isabela Pires-Dos-Santos, Raphael Mechoulam, Flávio Kapczinski, Benedito A. L. Fonseca, Danillo L. A. Esposito, Maristela Haddad Andraus, José Alexandre S. Crippa

**Affiliations:** ^1^ Department of Neuroscience and Behavior, Ribeirão Preto Medical School, University of São Paulo, Ribeirão Preto, Brazil; ^2^ National Institute for Science and Technology—Translational Medicine, Ribeirão Preto, Brazil; ^3^ Department of Pharmacology, Ribeirão Preto Medical School, University of São Paulo, Ribeirão Preto, Brazil; ^4^ University of Michigan Medical School, Ann Arbor, IN, United States; ^5^ Department of Psychiatry, Federal University of São Paulo, São Paulo, São Paulo, Brazil; ^6^ The Institute for Drug Research, School of Pharmacy, The Hebrew University of Jerusalem, Jerusalem, Israel; ^7^ Department of Psychiatry and Behavioural Neurosciences, McMaster University and St. Joseph’s Healthcare Hamilton, Hamilton, ON, Canada; ^8^ Department of Psychiatry, Faculty of Medicine, Graduate Program in Psychiatry and Behavioral Sciences, Universidade Federal do Rio Grande do Sul, Porto Alegre, Brazil; ^9^ Department of Internal Medicine, Ribeirão Preto Medical School, University of São Paulo, Ribeirão Preto, Brazil; ^10^ Chromatox Laboratory Ltd São Paulo, Brazil

**Keywords:** Cannabidiol, CBD, Anxiety, emotional exhausion, burnout, depression, healthcare worker (HCW), follow-up

## Abstract

**Objective:** To assess whether the effects of oral administration of 300 mg of Cannabidiol (CBD) for 28 days on mental health are maintained for a period after the medication discontinuation.

**Methods:** This is a 3-month follow-up observational and clinical trial study. The data were obtained from two studies performed simultaneously by the same team in the same period and region with Brazilian frontline healthcare workers during the COVID-19 pandemic. Scales to assess emotional symptoms were applied weekly, in the first month, and at weeks eight and 12.

**Results:** The primary outcome was that, compared to the control group, a significant reduction in General Anxiety Disorder-7 Questionnaire (GAD-7) from baseline values was observed in the CBD group on weeks two, four, and eight (Within-Subjects Contrasts, time-group interactions: F_1-125_ = 7.67; *p* = 0.006; η_p_^2^ = 0.06; F_1-125_ = 6.58; *p* = 0.01; η_p_^2^ = 0.05; F_1-125_ = 4.28; *p* = 0.04; η_p_^2^ = 0.03, respectively) after the end of the treatment.

**Conclusions:** The anxiolytic effects of CBD in frontline health care professionals during the COVID-19 pandemic were maintained up to 1 month after the treatment discontinuation, suggesting a persistent decrease in anxiety in this group in the real world. Future double-blind placebo-controlled clinical trials are needed to confirm the present findings and weigh the benefits of CBD therapy against potential undesired or adverse effects.

## Introduction

Anxiety disorders are the most prevalent mental illnesses globally, causing high social and economic costs (Stein et al., 2017). The usual pharmacological treatments for these conditions (anxiolytic and antidepressant medications) often have adverse effects and low efficacy (40%–60% of patients), with most patients failing to achieve complete remission (Bandelow et al., 2012). Thus, it is essential to develop novel treatment approaches for anxiety disorders.

Cannabidiol (CBD) is a nonpsychotomimetic constituent of the *Cannabis* plant, which has potential therapeutic properties across many neuropsychiatric disorders, such as anxiety, depression, psychotic disorder, epilepsy, cognitive disorder, addiction, and other indications like chronic pain, (Crippa et al., 2018) with a favorable safety and tolerability profile (Bergamaschi et al., 2011a). Several preclinical studies using different animal models have shown the potential anxiolytic properties of CBD (Guimarães et al., 1990; Moreira et al., 2006; Campos and Guimaraes, 2009; Soares et al., 2010; Campos et al., 2012). These properties have been found in humans after experimentally induced anxiety in healthy volunteers (Zuardi et al., 1993; Zuardi et al., 2017; Linares et al., 2018) and social phobia patients (Bergamaschi et al., 2011b; Crippa et al., 2011). A recent small clinical trial with Japanese teenagers also showed beneficial effects of CBD in this later disorder (Masataka, 2019). However, the number of clinical studies investigating the anxiolytic effects of CBD after repeated treatment is still limited.

Moreover, considering the increase in the medicinal use of CBD in recent years, the importance of extensive real-world studies in this area has been recently highlighted 16 (Schlag et al., 2021). We recently showed that CBD decreased emotional exhaustion/burnout and anxiety symptoms in healthcare workers (HCWs) in a 28-days intervention (Crippa et al., 2021) during the COVID-19 pandemic. This population is recognized as being at exceptionally high risk of developing anxiety symptoms (Sahebi et al., 2021). In a series of prospective cases of non-frontline health workers treated with CBD during the COVID-19 pandemic, we observed that the improvement of anxiety symptoms was sustained for more than 4 weeks after the treatment discontinuation (Pacheco et al., 2021). To verify in a controlled study if the anxiolytic effect of CBD persists for at least 4 weeks after its discontinuation, in the present paper, we conducted a 3-month follow-up of the clinical trial with frontline HCWs (Crippa et al., 2021) and compare this group with similar demographic and professional characteristics control group from another study conducted in parallel at the same period (Osório et al., 2021).

## Materials and methods

### Design

The data of this study were obtained from two studies with frontline HCWs involved with COVID-19 treatment performed simultaneously by the same team in the same period and region. Partial results of these studies have been published (Crippa et al., 2021) (Osório et al., 2021). One study was a clinical trial, two-arm, parallel-group, unblinded with evaluator blinded, to test the efficacy of oral CBD to prevent or reduce emotional distress in HCWs dealing with COVID-19 patients (Crippa et al., 2021). The other was an observational study to assess and monitor emotional distress among health workers providing care to patients with COVID-19 (Osório et al., 2021). The two studies were approved by the Institutional Review Board (Process N^o^ 4.190.338 and 4.032.190).

### Summary of the reference studies

The participants of the two studies were recruited between May to November 2020, during the first wave of COVID-19 in Brazil, *via* institutional advertising, email, and social media. The inclusion criteria were being healthcare workers (nurses, physicians, physical therapists, occupational therapists, speech therapists, psychologists, social workers, and nutritionists) of both sexes involved in the treatment of COVID-19 patients and providing their informed consent. The exclusion criteria in the clinical trial were the use of any medication with potential interactions with CBD, a history of undesirable reactions to CBD or other cannabinoids, pregnancy, and belonging to COVID-19 risk groups.

Participants of the two studies completed an online survey with scales applied weekly, in the first month, and at weeks 8–12. The primary outcome measure includes the Generalized Anxiety Disorder Questionnaire—[GAD-7] (Moreno et al., 2016), a 7-item self-report instrument that screens anxiety-associated symptoms rated on a three-point scale ranging from 0 (never) to 3 (almost every day). The secondary outcome measures include the Patient Health Questionnaire-9 [PHQ-9] (de Lima Osório et al., 2009), a 9-item self-report instrument intended to assess depression indicators, rated from 0 (“never”) to 3 (“almost every day”); the Abbreviated Maslach Burnout Inventory-subscale emotional exhaustion [aMBI]) (Carlotto and Camara, 2007), a four items that evaluate emotional depletion due to job demand on a seven-point Likert scale, ranging from 0 (“never”) to 6 (“every day”); the Posttraumatic Stress Disorder Checklist – 5 (PCL-5) (Osório et al., 2017), a self-report instrument used to assess symptoms of posttraumatic stress disorder using the criteria established by the DSM-5; and a questionnaire about their demographic and professional characteristics, and personal clinical variables associated with mental health. These data were automatically stored in the RedCap platform.

In the clinical trial, in addition to the follow-up with the rankings, one group received oral CBD (99.6% purity: PurMed Global, Delray Beach, Florida, United States ) dissolved in medium-chain triglyceride oil for the first 4 weeks (150 mg twice a day). The dose of CBD was defined based on previous evidence showing that 300–400 mg/d doses promote anxiolytic effects within good safety and tolerability standards (Zuardi et al., 2017; Crippa et al., 2018; Linares et al., 2018). The treatment safety of CBD was assessed with a modified version of the UKU side effect rating scale of the Scandinavian Society of Psychopharmacology (Lingjaerde, 1987), highlighting the most common adverse effects of CBD, the “CBD Adverse Effects Scale” (CARE Scale). In these participants, we evaluated the plasma levels of CBD and general laboratory exams in samples collected at baseline and on days 7, 14, 21, and 28.

### Procedure of the present study

We compared two groups with data extracted from participants of the referred studies (Crippa et al., 2021; Osório et al., 2021). The healthcare workers from the clinical trial (Crippa et al., 2021), who received CBD during the first month of follow-up, formed one group (CBD group). The other group consisted of healthcare workers from the observational study (Osório et al., 2021), who did not receive a pharmacological intervention (Control group-CG). All healthcare workers worked in institutions located in the northeast region of the state of São Paulo, Brazil (100 km around the city of Ribeirão Preto).

Participants were paired allocated into the two groups by a researcher blinded to the individual results of the rating scales. Considering that the dropouts were around twice as prominent in the study without CBD (Osório et al., 2021), the participants were randomized in a 1:2 (CBD:CG) ratio concerning sex, age, and profession (Nursing or other). Assuming an effect size (Cohen’s d) of 0.1, level of significance of 0.05, and a statistical power of 0.8, we estimated that 142 participants (around 71 per group) who completed the 3 months were adequate for detecting a small effect. To obtain this number of participants at the end of 12 weeks, we selected at baseline 100 and 200 subjects in the CBD group (30% of dropouts) and Control group (65% dropouts), respectively.

### Statistical analysis

Clinical and demographic data comparing CBD and Control groups at baseline, and after 12 weeks, from subjects that completed or not completed the 12-weeks observational period, were analyzed using the t-test for continuous data and the Fisher’s Exact test for nominal data. Data from the rating scales were analyzed with a repeated-measures analysis of variance (repeated-measures ANOVA) with factors time, group, and time × group interaction. The degrees of freedom of the repeated factor were corrected with Huynh-Feldt epsilon when sphericity conditions were not met. Within-subjects contrasts with a significant time × group interaction assessed the differences between groups in each measured compared to the baseline.

In the group treated with CBD during the first 4 weeks, we compared the percentage of participants that showed a reduction in clinical anxiety (cutoff score of 10 points or greater on GAD-7) in the four quartiles of the CBD plasma level. To assess whether the decrease in clinical anxiety was associated with plasma levels of CBD, we used the bipolar logistic regression (fourth quartile compared with the other quartiles). The significance level was set at *p* < 05.

## Results

### Participants

At baseline and after 12 weeks of follow-up, there was no significant difference between the CBD and Control groups concerning sex, age, occupation, psychiatric diagnosis, psychological treatment, and psychiatric medication. In addition, the same characteristics did not differ significantly between those who completed or not the 12 weeks of follow-up. Demographic and clinical characteristics are summarized in Table 1.

**TABLE 1:** Demographic and Clinical Characteristics of Participants.

Characteristic	Baseline			Completed 3 months			Comparison of whether they completed 3 months		
	CBD	Control	p	CBD	Control	p	Yes	No	p
	**N = 100**	**N = 200**		**N = 71**	**N = 79**		**N = 150**	**N = 150**		
**Sex N (%)**									
Female	79 (79)	153 (76.5)	0.626	56 (78.9)	63 (79.7)	0,895	119 (79.3)	113 (75.3)	0.408
Male	21(21)	47 (23.5)		15 (21.1)	16 (20.3)		31 (20.7)	37 (24.7)	
**Age (years) Mean (SD)**									
	34.45 (7.30)	34.75 (9.56)	0.783	34.51 (7.36)	35.6 (10.16)	0.458	35.08 (8.93)	34.22 (8.79)	0.401
**Occupation – No. (%)**									
Nurse	44 (44)	105 (52.5)	0.165	30 (42.3)	36 (45.6)	0,136	73 (48.7)	76 (50.7)	0.729
Other	56 (56)	95 (47.5)		41 (57.7)	43 (54.4)		77 (51.3)	74(49.3)	
**Psychiatric diagnosis No. (%)**									
Yes	22(22)	52 (26)	0.397	16 (22.5)	26 (32.9)	0,158	42 (28)	32 (21.1)	0.16
No	78 (78)	148 (74)		55(77.5)	53 (67.1)		108 (72)	120 (78.9)	
**Psychological treatment – No. (%)**									
Yes	43 (43)	86 (43)	1.0	27 (38)	39 (49.4)	0,162	66 (44)	63 (42)	0.726
No	57 (57)	114 (57)		44 (62)	40 (50.6)		84 (56)	87 (58)	
**Psychiatric medication No. (%)**									
Yes	20 (20)	44 (22)	0.630	16 (22.5)	26 (32.9))	0,158	36 (24)	28 (18.67)	0.236
No	80(80)	156 (78)		55 (75.5)	53 (67.1)		114 (76)	122 (81.33)	

### Primary outcome

**FIGURE 1:**
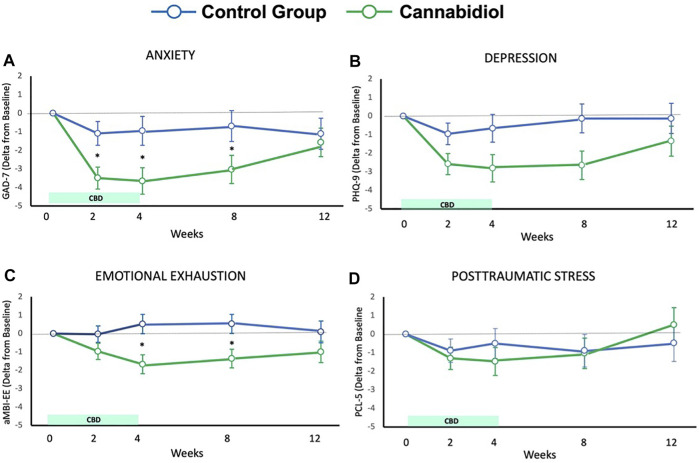
Results for Anxiety **(A)**, depression **(B)**, emotional exhaustion **(C)**, and posttraumatic stress **(D)**.

The ANOVA repeated measures of GAD-7 scores showed a significant effect of time (F_3.28-410.18_ = 8.18; *p* < 0.001; η_p_^2^ = 0.06), group (F_1-125_ = 4.69; *p* = 0.03; η_p_^2^ = 0.04), and time-group interaction (F_3.28-410.18_ = 3.51; *p* = 0.01; η_p_^2^ = 0.03). Compared to Control group, a significant reduction in delta score concerning baseline values was observed in the CBD group on weeks two, four, and eight (Within-Subjects Contrasts, time-group interactions: F_1-125_ = 7.67; *p* = 0.006; η_p_^2^ = 0.06; F_1-125_ = 6.58; *p* = 0.01; η_p_^2^ = 0.05; F_1-125_ = 4.28; *p* = 0.04; η_p_^2^ = 0.03, respectively). Figure 1A showed the delta score, concerning baseline values in weeks 2,4,8, and 12. The repeated measures ANOVA of the GAD-7 scores showed a significant effect of time only for the CBD group (F_3.46-235.05_ = 10.56; *p* < 0.001; η_p_^2^ = 0.13). The differences between the basal and the other time points are shown in Figure 1A.

**FIGURE 2:**
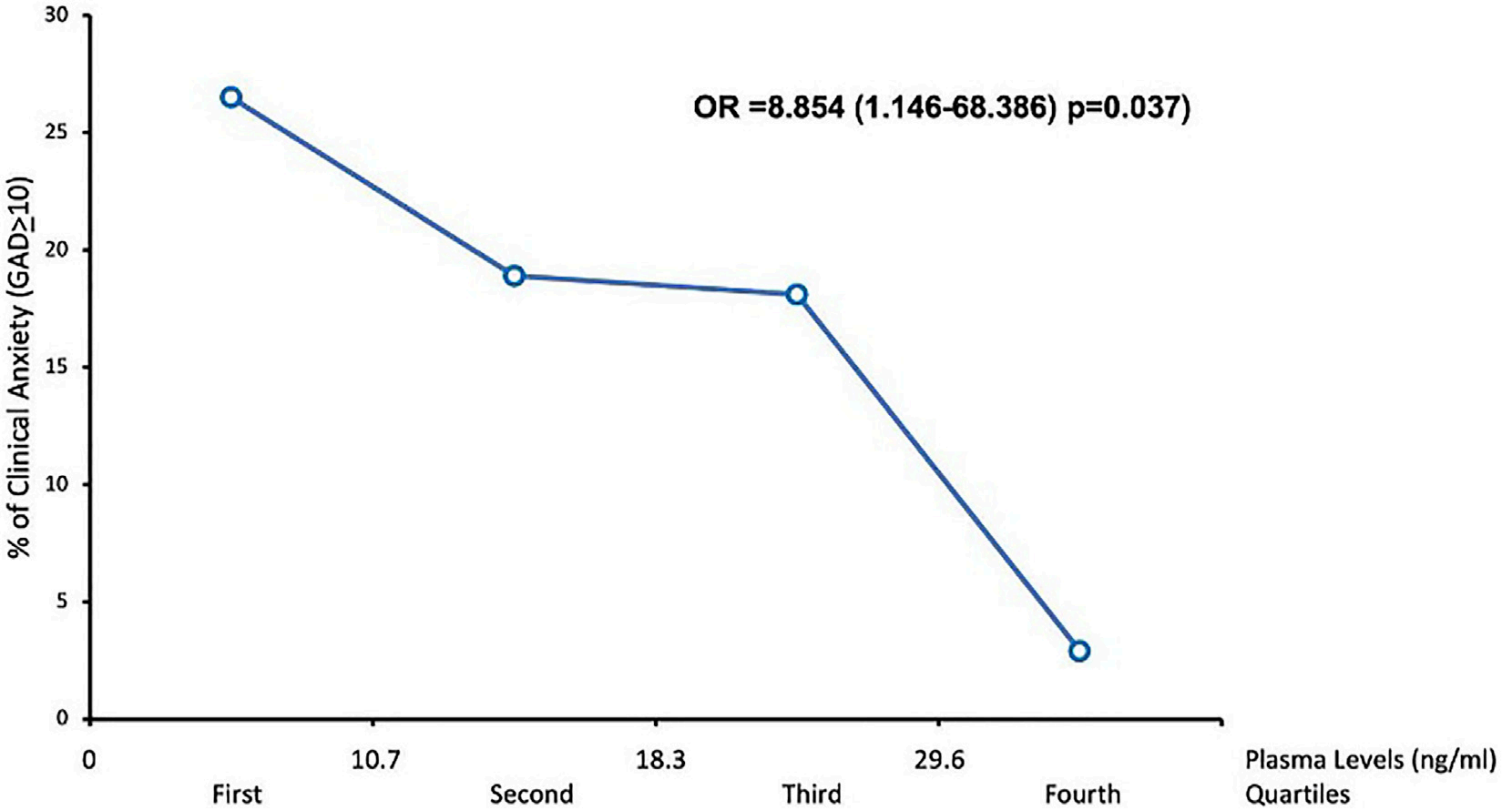
Association between CBD plasma levels and anxiety symptoms.

In the group treated with CBD during the first 4 weeks, the percentage of participants that showed a reduction in clinical anxiety in the four quartiles of the CBD plasma level was shown in Figure 2. CBD plasma levels were significantly associated with the reduction in clinical anxiety (Odd ratio = 8.854; Confidence interval = 1.146-68.386; *p* = 0.037).

### Secondary outcome

The delta score concerning baseline values of the scales PHQ-9, aMBI, and PCL-5 was shown in Figure 1 as well.

There were significant effects of time (F_3.31-443.49_ = 5.37; *p* < 0.001; η_p_^2^ = 0.04), group (F_1-134_ = 4.85; *p* = 0.03; η_p_^2^ = 0.04), and tendency to significant effect of time × group interaction (F_3.31-443.49_ = 2.09; *p* = 0.08; η_p_^2^ = 0.02), on PHQ-9 scores. The Within-Subjects Contrasts showed a significant effect of time × group interaction on weeks two (F_1-134_ = 4.02; *p* = 0.04; η_p_^2^ = 0.03), four (F_1-134_ = 4.23; *p* = 0.04; η_p_^2^ = 0.03), and eight (F_1-134_ = 5.32; *p* = 0.02; η_p_^2^ = 0.04), with a lower value in CBD group (Figure 1B). The repeated measures ANOVA of the PHQ-9 scores showed a significant effect of time only for the CBD group [F_3.36-228.23_ = 5.62; *p* = 0.001; η_p_^2^ = 0.08]. The differences between the basal and the other time points are shown in Figure 1B.

Concerning aMBI scores, there were significant effects of group (F_1-133_ = 6.04; *p* = 0.02; η_p_^2^ = 0.04), and time × group interaction (F_3.57-474.03_ = 3.19; *p* = 0.02; η_p_^2^ = 0.02), but no significant effect of time (F_3.57-474.03_ = 3.19; *p* = 0.02; η_p_^2^ = 0.02). The Within-Subjects Contrasts showed a significant effect of time × group interaction on weeks four (F_1-133_ = 8.73; *p* = 0.004; η_p_^2^ = 0.06), and eight (F_1-133_ = 6.66; *p* = 0.01; η_p_^2^ = 0.05), with a lower value in CBD group. (Figure 1C). The repeated measures ANOVA of the aMBI scores showed a significant effect of time only for the CBD group (F_3.18-216.23_ = 3.57; *p* = 0.007; η_p_^2^ = 0.05). The differences between the basal and the other time points can be seen in Figure 1C.

The repeated measures ANOVA of the PCL scores did not show a significant effect of time (F_3.19-414.59_ = 2.08; *p* = 0.1; η_p_^2^ = 0.02), group (F_1-130_ = 0.02; *p* = 0.89; η_p_^2^<0.001), and time-group interactions (F_3.19-414.59_ = 0.85; *p* = 0.47; η_p_^2^ = 0.006). The repeated measures ANOVA of the PCL scores showed a significant effect of times only for the CBD group (F_3.41-235.10_ = 2.61; *p* = 0.036; η_p_^2^ = 0.04). The differences between the basal and the other time points are shown in Figure 1D.

### Safety of cannabidiol treatment

The serious adverse events observed during the 4 weeks of CBD treatment were increased hepatic enzymes greater than three times the upper limit (4%), none with total serum bilirubin levels greater than two-fold, and reports of skin erythema diagnosed as pharmacodermia (4%) (Souza et al., 2022). All cases fully recovered after CBD discontinuation. The most common adverse effects were somnolence (19%), diarrhea (15%), increased appetite (11%), and fatigue (10%).

## Discussion

This observational and clinical trial study combination follow-up showed that CBD’s beneficial effects on mental health were maintained after 1 month of its discontinuation in frontline HCWs during the COVID-19 pandemic. Compared to the control group, these effects included a reduction in anxiety, depressive, and emotional exhaustion/burnout symptoms measured by the GAD-7, PHQ-9, and aMBI scores, respectively. Furthermore, there was a statistically significant association between the higher CBD plasma level and the lower number of participants with scores indicative of anxiety (GAD-7 score >9 points. Overall, existing preclinical and clinical evidence support a potential role for CBD as a novel treatment for anxiety disorders (Crippa et al., 2018). The clinical evidence to date is mostly based on acute single-dose studies. Therefore, the present research contributes by showing not only the beneficial effects of daily CBD administration for 28 days but also that these effects are maintained over a similar period. Since the half-life of CBD after chronic oral administration is between two and 5 days (Millar et al., 2018), the maintenance of the anxiety and emotional exhaustion attenuation could not be attributed to the plasma level of the drug. Instead, one possible explanation is that a period of less anxiety at work induced by CBD would transiently change their aversive memory concerning the work by their effects of enhancing the extinction (Das et al., 2013) or interfering with the reconsolidation of traumatic memories (Bolsoni et al., 2022). However, these effects did not occur in depression and post-traumatic stress disorder. The discontinuation of CBD after 4 weeks did not induce withdrawal signs.

Cannabis products are widely employed worldwide, and their use has been illegalized in several countries (Bonomo et al., 2018)^.^ After legalizing medicinal and recreational cannabis in some countries, its use highly increased (Wen et al., 2015; Martins et al., 2016; Hasin et al., 2017). CBD has gained special attention for its therapeutic potency for several physical and mental health conditions (Sholler et al., 2020). Anxiety is regularly one of the most frequent conditions for which patients use cannabis (Schlag et al., 2021), particularly pure CBD products (Leas et al., 2020) There are other anxiolytic drugs; however, these can have some adverse effects that are even more harmful to healthcare professionals on the front lines of caring for COVID-19 patients. Among these, we can mention sedation, cognitive dysfunction, decreased performance of benzodiazepines (Lader, 2014) and latency to onset of anxiolytic effects with a possible increase in anxiety, at the beginning of treatment, with antidepressants (Moreno et al., 1999). Therefore, considering the increase in the medicinal use of CBD in recent years, extensive real-world studies on this area are urgently needed.

Treatment with cannabidiol was associated with few reported cases of serious AEs, which resolved after drug discontinuation. Still, their presence highlights the need for close clinical monitoring (especially liver function testing) of patients receiving CBD therapy (Sholler et al., 2020). To our knowledge, this is the first study to assess the effects of CBD even after the medication discontinuation. Thus, the present work results could significantly impact the medicinal cannabinoids used worldwide. However, more clinical trials are needed to assess the long-term effects of Cannabidiol.

### Limitations

The main limitation is that the samples compared, although with similar demographic and professional characteristics, are from different studies. An ideal protocol would include a placebo-controlled treatment to discard a putative placebo effect in the CBD group. However, the main objective of this study was to evaluate the duration of the effects observed after the treatment discontinuation, and in the 2 months after CBD treatment, the two groups had the same protocol, only the monitoring with self-evaluated scales. In addition, the much lower dropout rate in the control (65%) compared to the CBD-treated group (30%) could reflect a more significant beneficial effect observed in this group. Since the survey was online, no urine tests were made to rule out substance use.

## Conclusion

This observational and clinical trial study combination follow-up showed that the beneficial effects on anxiety, emotional exhaustion/burnout, and depressive symptoms observed among frontline health care professionals working with patients with COVID-19 after 28 days of daily CBD administration were maintained for up to a month after the treatment discontinuation. This study meets the recently highlighted need for extensive real-world studies on CBD’s potential medicinal use. Future double-blind placebo-controlled clinical trials are needed to assess the CBD long-term effects and confirm the present findings.

## Data Availability

The original contributions presented in the study are included in the article, further inquiries can be directed to the corresponding author.
